# To analyze the relationship between gut microbiota, metabolites and migraine: a two-sample Mendelian randomization study

**DOI:** 10.3389/fmicb.2024.1325047

**Published:** 2024-04-16

**Authors:** Kang Qu, Ming-Xi Li, Lin Gan, Zi-Ting Cui, Jia-Jia Li, Rong Yang, Ming Dong

**Affiliations:** Department of Neurology and Neuroscience Center, The First Hospital of Jilin University, Changchun, China

**Keywords:** migraine, gut microbiota, metabolite, gut-brain axis, causal inference, Mendelian randomization

## Abstract

**Background:**

It has been suggested in several observational studies that migraines are associated with the gut microbiota. It remains unclear, however, how the gut microbiota and migraines are causally related.

**Methods:**

We performed a bidirectional two-sample mendelian randomization study. Genome-wide association study (GWAS) summary statistics for the gut microbiota were obtained from the MiBioGen consortium (*n* = 18,340) and the Dutch Microbiota Project (*n* = 7,738). Pooled GWAS data for plasma metabolites were obtained from four different human metabolomics studies. GWAS summary data for migraine (cases = 48,975; controls = 450,381) were sourced from the International Headache Genetics Consortium. We used inverse-variance weighting as the primary analysis. Multiple sensitivity analyses were performed to ensure the robustness of the estimated results. We also conducted reverse mendelian randomization when a causal relationship between exposure and migraine was found.

**Results:**

*LachnospiraceaeUCG001* (OR = 1.12, 95% CI: 1.05–1.20) was a risk factor for migraine. *Blautia* (OR = 0.93, 95% CI: 0.88–0.99), *Eubacterium* (*nodatum* group; OR = 0.94, 95% CI: 0.90–0.98), and *Bacteroides fragilis* (OR = 0.97, 95% CI: 0.94–1.00) may have a suggestive association with a lower migraine risk. Functional pathways of methionine synthesis (OR = 0.89, 95% CI: 0.83–0.95) associated with microbiota abundance and plasma hydrocinnamate (OR = 0.85, 95% CI: 0.73–1.00), which are downstream metabolites of *Blautia* and *Bacteroides fragilis*, respectively, may also be associated with lower migraine risk. No causal association between migraine and the gut microbiota or metabolites was found in reverse mendelian randomization analysis. Both significant horizontal pleiotropy and significant heterogeneity were not clearly identified.

**Conclusion:**

This Mendelian randomization analysis showed that *LachnospiraceaeUCG001* was associated with an increased risk of migraine, while some bacteria in the gut microbiota may reduce migraine risk. These findings provide a reference for a deeper comprehension of the role of the gut–brain axis in migraine as well as possible targets for treatment interventions.

## Introduction

Recurrent migraine attacks are a debilitating neurological disease that is the second leading cause of disability (GBD 2016 Disease and Injury Incidence and Prevalence Collaborators, 2017; Dodick, 2018). It is thought to affect over 1 billion individuals globally and imposes a substantial economic burden on individuals and society (Ashina et al., 2021). To date, the causes and pathology of migraines are still poorly understood. Evidence has indicated that background genetic and environmental factors influence the onset and progression of migraine (Goadsby et al., 2017). Genetic studies have suggested that approximately half of the individual risk variations for migraine are heritable, underscoring the genetic contribution to the etiology of migraine (Piane et al., 2007).

In addition to genetics and environment, the microbiota–gut–brain axis has been hypothesized as a key mechanism through which gut microbiota might trigger migraine attacks (Arzani et al., 2020). The microbiota–gut–brain axis is currently a subject of intense interest. Research focuses on studying the relationship between gut microbiota and hosts and describes the flow of information between the brain and gut microbiota (Cryan et al., 2019; Socała et al., 2021). Imbalances in the gut microbiota abundance may affect the release of neurotransmitters and inflammatory mediators, such as glutamate, calcitonin gene-related peptide, and interleukin (IL)-1β, which might increase the risk of migraine (Arzani et al., 2020; Crawford et al., 2022). Therefore, balancing the abundance of gut microbiota, such as by probiotic supplementation, is thought to be a promising and intriguing therapeutic target (Arzani et al., 2020).

Several observational studies have found notable differences in the makeup and quantity of certain resident microorganisms in people with migraine compared to those without, including increased amounts of *Alcaligenes*, *Clostridium coccoides*, and *Eggerthella lenta* and a decreased proportion of *Faecalibacterium prausnitzii* and *Bifidobacterium adolescentis* (Chen et al., 2019; Georgescu et al., 2019; Kopchak et al., 2022; Kopchak and Hrytsenko, 2022). Additionally, it was shown that the frequency and intensity of migraine attacks were negatively associated with the abundance of *Alcaligenes*; *Eggerthella lenta* levels were positively correlated with visual analog scores in people with migraine (Kopchak et al., 2022; Kopchak and Hrytsenko, 2022). The categories in the oral microbiome of people with migraine and people without differed in a study using 16S rRNA gene sequencing (Jiang et al., 2021). The frequency and severity of migraine attacks were improved by multiple probiotic formulations according to observational research including 1,020 people with migraine (Straube et al., 2018). However, the conclusions of several published studies have been contradictory (De Roos et al., 2017; Chen et al., 2019; Martami et al., 2019). The results of a meta-analysis, which included only three randomized controlled studies, suggested that probiotic supplementation may not significantly improve migraine frequency or severity (Parohan et al., 2022).

In observational study, confounding variables including age, environmental circumstances, and way of life could easily bias the connection between the gut microbiota and migraine (Rinninella et al., 2019). At the same time, considering that most observational studies are designed as case–control studies, it is difficult to judge the chronological order of exposure factors and disease outcomes or to clarify causal relationships between them (Davey Smith and Hemani, 2014). Randomized controlled trials serve a dual purpose by not only exploring causality but also providing valuable insights into the efficacy of treatments and prognosis. Greater benefits to public health will result from investigating the associations between the gut microbiota and migraine, which will help with primary migraine prevention.

Mendelian randomization (MR) using genetic variants of exposure as instrumental variables (IVs) is a novel approach investigating exposure factors and illness outcomes (Bowden et al., 2019). There is reduced confounding bias in MR investigations because genetic variations are allocated at random and appear before diseases do (Lin et al., 2021). In two-sample MR analysis, exposure and outcome data are derived from two independent genome-wide association studies (GWAS) to avoid bias caused by samples from the same population (Xu et al., 2021). In the present study, we applied a two-sample MR design to evaluate the association between gut microbiota and migraine, according to the STROBEMR guidelines (Skrivankova et al., 2021).

## Methods

### Assumptions for MR and study design

**Figure 1:**
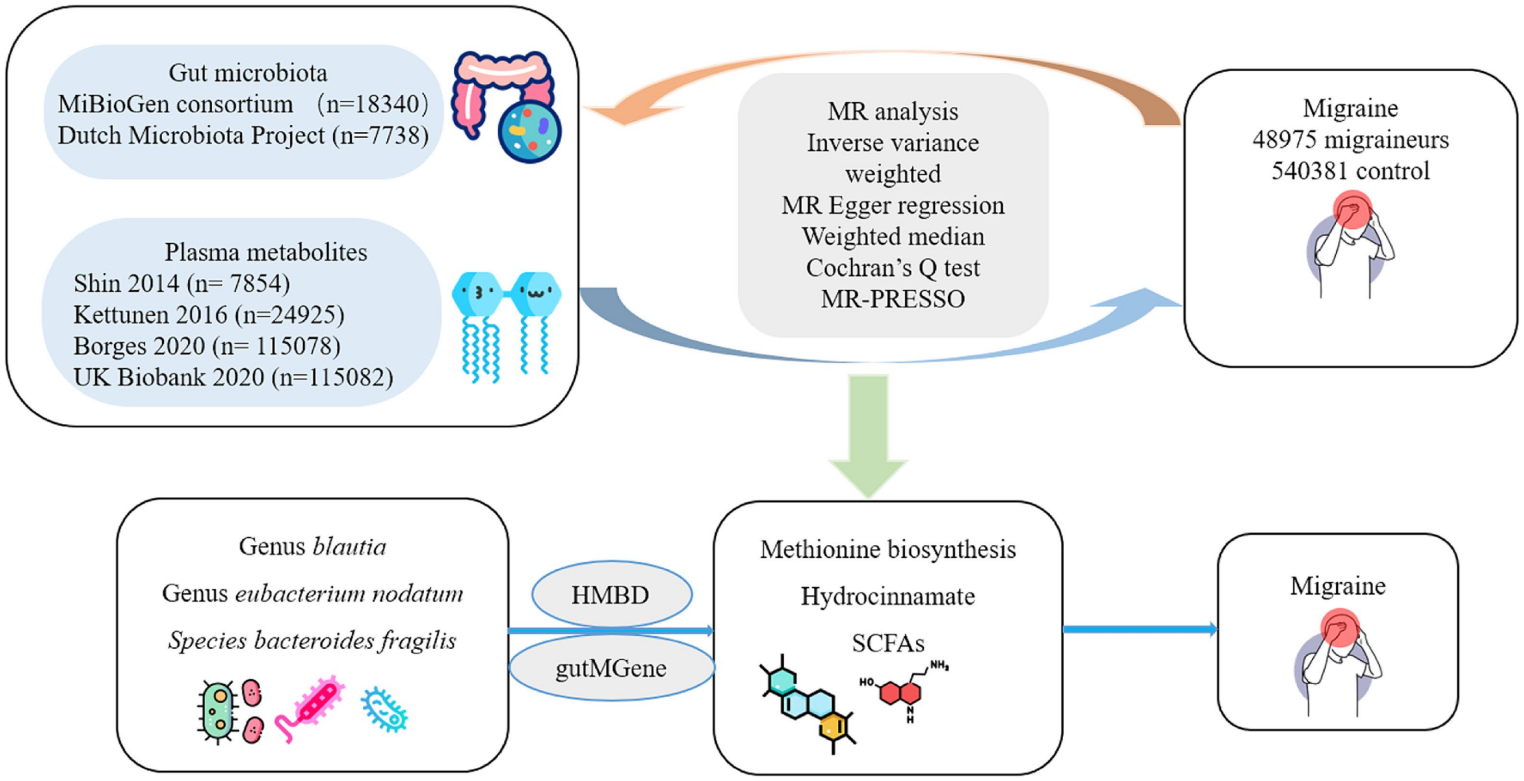
Flowchart of the study.

The IVs in the MR analysis must meet the following three assumptions (Davey Smith and Hemani, 2014): (1) IVs that are closely related to the gut microbiota, (2) IVs that are independent of confounding factors (affecting gut microbiota and migraine), and (3) IVs that affect migraine only through the gut microbiota and not through other factors. Figure 1 presents a flowchart of the MR analyses conducted in this study.

### Genome-wide association study data

GWAS summary statistics for the gut microbiota were obtained from the MiBioGen consortium (Kurilshikov et al., 2021; Consortium M, 2022). This study included 24 cohorts with a total of 18,340 individuals, making it the largest GWAS meta-analysis of gut microbiota to date, with 13,266 participants of European ancestry. Notably, given the substantial representation of participants with European ancestry, this dataset is suitable for MR analysis within European populations. The microbial composition of the participants was analyzed. To ensure that host genetic variation correlated with the abundance of bacterial taxa, the mean abundance level of bacterial taxa had to be greater than 1%. This decision was based on the proportion of taxa present in the samples and microbial quantitative trait loci mapping analysis. Direct taxonomic binning was used to classify the taxa. The bacterial taxa included in this study included phylum (*n* = 9), class (*n* = 16), order (*n* = 20), family (*n* = 32) and genus (*n* = 119), respectively. Fifteen bacterial taxa (unidentified family or genus) without specific species names were omitted from the investigation. Another GWAS summary dataset for gut microbial taxa was obtained from the Dutch Microbiota Project, which included 7,738 individuals of European ancestry (Lopera-Maya et al., 2022). A total of 207 microbial taxa (5 phyla, 10 classes, 13 orders, 26 families, 48 genera, 105 species) and 205 functional pathways were included in this study.

Given the role of gut metabolites as a bridge between the microbiota and host, microbes may influence the activity and function of the host nervous system by altering metabolite levels. We included data from four human metabolomic GWAS conducted in individuals of European ancestry (Shin et al., 2014; Kettunen et al., 2016; Hemani et al., 2018; Richardson et al., 2022). One study, which included 7,824 participants, analyzed 452 plasma metabolites, and another study, which included 24,925 participants, analyzed 123 metabolites. The two remaining studies investigated 249 metabolites and included 115,078 and 115,082 participants each. First, plasma metabolites associated with migraine were identified. Next, the Human Metabolome Database (Wishart et al., 2022) and gutMGene (Cheng et al., 2020) were used to identify whether the plasma metabolites identified in the first step were associated with the gut microbiota.

The summary-level GWAS data for migraine were obtained from a meta-analysis of the GWAS of participants of European ancestry from the International Headache Genetics Consortium (IHGC). The acquisition of the data was approved through a direct application and material transfer agreement. This study was conducted by Hautakangas et al. (Hautakangas et al., 2022) and included data from five studies. A total of 102,048 migraine patients and 771,257 controls were included. In the present study, the data used were from 48,975 people with migraine and 540,381 control individuals from four studies (23andMe was not included because of the strict participant privacy protections in this cohort). Migraine cases were identified based on self-reported data or the International Classification of Headache Disorders. The cases included in this analysis were adjusted for sex, age, and ancestry. The original article has the rest of the information, such as demographic information (Hautakangas et al., 2022). Details of the cohorts included in both GWAS studies are available (Kurilshikov et al., 2021; Hautakangas et al., 2022). There was a limited amount of sample overlap between two GWAS data included in this study (<2%). Moreover, two-sample Mendelian analysis can be applied to large datasets with overlapping samples (Minelli et al., 2021). Detailed GWAS information is presented in Supplementary Table S1.

### Instrumental variables selection

Screening criteria for the IVs included the following. A significance level of a single-nucleotide polymorphism (SNP) of *p* < 1.0 × 10^−5^ was considered a candidate IV. This threshold aligns with similar criteria used in published studies (Sanna et al., 2019). To ensure the independence of each IV, linkage disequilibrium was set at *R*^2^ < 0.001 and a clumping distance of 10,000 kb, using the European-based 1,000 Genome Projects in the R package. Minor allele frequencies ≤0.01 may represent rare variants, and SNPs with minor allele frequencies ≤0.01 are controversial in genetic association analyses, so these were excluded. F-statistics were calculated according to the calculation formula in the previous literature (Pierce et al., 2011). Weak IVs with F-statistics less than 10 were excluded to prevent violation of the first assumption of MR. Palindromic SNPs with intermediate allele frequencies were also removed. Finally, the PhenoScanner^1^ (PhenoScanner, n.d.), a platform capable of querying information related to genotypes and phenotypes, was employed. Additional checks to exclude potential associations between the included IVs and currently recognized risk factors for migraine, such as tobacco use, caffeine consumption, diastolic blood pressure, systolic blood pressure, vitamin D, depression, and anxiety, were conducted (Guo et al., 2020; Yuan et al., 2022; Zheng et al., 2023). If present, SNPs significantly associated with these potential confounders at genome-wide significance (*p* < 1.0 × 10^−5^) were removed.

### Statistical analysis

Three different methods were used for the bidirectional two-sample MR analysis: random-effect inverse-variance weighting (IVW), MR-Egger, and weighted median (Burgess et al., 2013; Bowden et al., 2015, 2016). IVW is a meta-analysis of Wald ratios for each SNP. Without horizontal pleiotropy, the IVW results are unbiased and serve as the primary outcome of the analysis. When horizontal pleiotropy is present, the MR-egger method can be applied, but it requires that the pleiotropy effect be independent of the variant–exposure association. The weighted median was used to calculate the median effect of SNPs and allowed for invalid tools, provided that the proportion of invalid IVs was less than 50% in the MR analysis. Cochran’s Q test was used to assess heterogeneity. It was assumed that there was no significant heterogeneity among the included IVs when the *p*-value of the Q test was greater than 0.05. The intercept term of the MR-Egger regression can be used to determine whether there is horizontal pleiotropy. If the intercept is not statistically different from zero, no significant horizontal pleiotropy is suggested (Bowden et al., 2015). The MR-Pleiotropy RESidual Sum and Outlier (MR-PRESSO) analysis was employed to assess the presence of outliers and mitigate the effects of horizontal pleiotropy (Verbanck et al., 2018). Outliers that potentially influenced the results were systematically removed until the global test *p*-value exceeded 0.05 (Wootton et al., 2020). Power calculations for MR were performed using the mRnd website^2^ (Burgess, n.d.).

To avoid the possible interference of reverse causation in the causal inference between gut microbiota and migraine, reverse MR analysis should be considered. The present reverse MR analysis used migraine as the exposure and gut microbiota as the outcome. The methods and procedures used in the reverse Mendelian analysis were consistent with those used in the forward Mendelian analysis.

Association effects were described as odds ratios (OR) with 95% confidence intervals (CI). This result was considered robust when the estimated effects of the three methods of analysis were the same and at least the IVW method estimate was significant. An approach called false discovery rate (FDR) control was used to prevent bias caused by multiple testing. The q-value (corrected *p*-value) was calculated with an FDR of q-value <0.05. A suggestive association between the gut microbiota and migraine was considered when *p* < 0.05 but *q* ≥ 0.05.

R software was used to conduct all statistical analyses (Version 4.2.2; R Foundation for Statistical Computing, Vienna, Austria). The R package for MR analysis consisted of the TwoSampleMR (version 0.5.6), MR-PRESSO (version 1.0), and qvalue R packages (Storey and Tibshirani, 2003; Hemani et al., 2017; Verbanck et al., 2018).

## Results

### Gut microbiota (MiBioGen) and migraine

The selection standards for IVs ultimately resulted in the identification of 2,120 SNPs as IVs associated with 196 bacterial taxa (Supplementary Table S2). There was no bias driven by weak IVs in the MR analysis, as each of the included SNPs had an F-statistic greater than 10.

**Figure 2:**
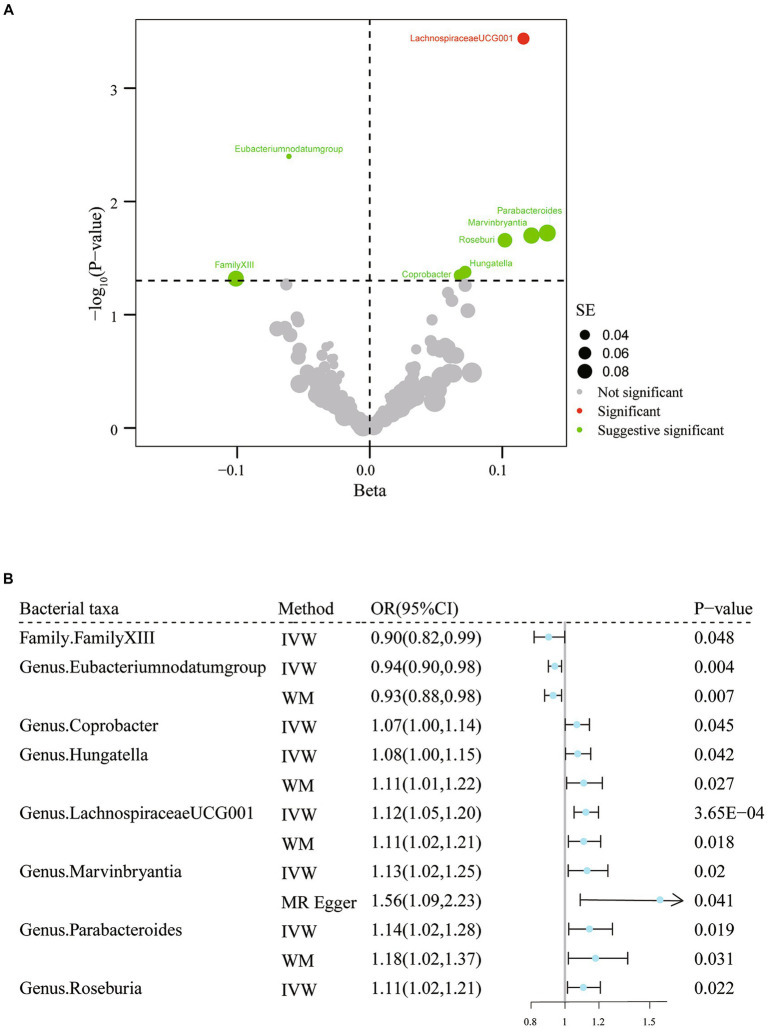
Results of IVW analysis of gut microbiota (MiBioGen) and migraine. **(A)** The volcano plot shows the association between 196 gut microbiota and migraine risk. The X-axis represents the β value and the Y-axis shows the logarithmic *p*-value in base 10. The red dots indicate the p-value <0.05 and false discovery rate < 0.05. Green dots indicate *p*-values <0.05 but the false discovery rate > 0.05. **(B)** Forest plot results for association estimates of gut microbiota and migraine. CI, confidence interval; IVW, inverse-variance weighted; WM, weighted median; OR, odds ratio; SE, standard error.

To determine whether any bacterial taxa were associated with migraine at the phylum, class, order, family, and genus levels, we first preprocessed the data using the IVW approach (Figure 2; Supplementary Table S3). We found that family *Family XIII* (OR = 0.90, 95% CI: 0.82–1.00, *p* = 0.048) and genus *Eubacterium* (*nodatum* group) (OR = 0.94, 95% CI: 0.90–0.98, *p* = 4.00 × 10^−3^) were associated with a lower risk of migraine, while genus *Coprobacter* (OR = 1.07, 95% CI: 1.00–1.14, *p* = 0.045), genera Hungatella (OR = 1.08, 95% CI: 1.00–1.15, *p* = 0.04), *LachnospiraceaeUCG001* (OR = 1.12, 95% CI: 1.05–1.20, *p* = 3.65× 10^−4^), *Marvinbryantia* (OR = 1.13, 95% CI: 1.02–1.25, *p* = 0.02), *Parabacteroides* (OR = 1.14, 95% CI: 1.02–1.28, *p* = 0.02), and *Roseburia* (OR = 1.11, 95% CI: 1.02–1.21, *p* = 0.02) were linked to a higher risk of migraine (Figure 2; Supplementary Table S4). When FDR was considered, only the genus *LachnospiraceaeUCG001* (*q* = 0.03) remained significantly associated with migraine. The remaining associations should only be considered suggestive (all *q* > 0.05). Regarding the sensitivity analysis, the estimated results of the other two analysis methods were in the same direction as those of the IVW method (Supplementary Table S4). Additionally, there was no strong reason to suspect horizontal pleiotropy based on the results of Cochran’s Q test, the MR-Egger regression intercept test, and the MR-PRESSO analysis (Supplementary Table S4). Thus, there was a causal relationship between the genus *Lachnospiraceae UCG 001* and migraine.

### Gut microbiota (Dutch microbiota project) and migraine

**Figure 3:**
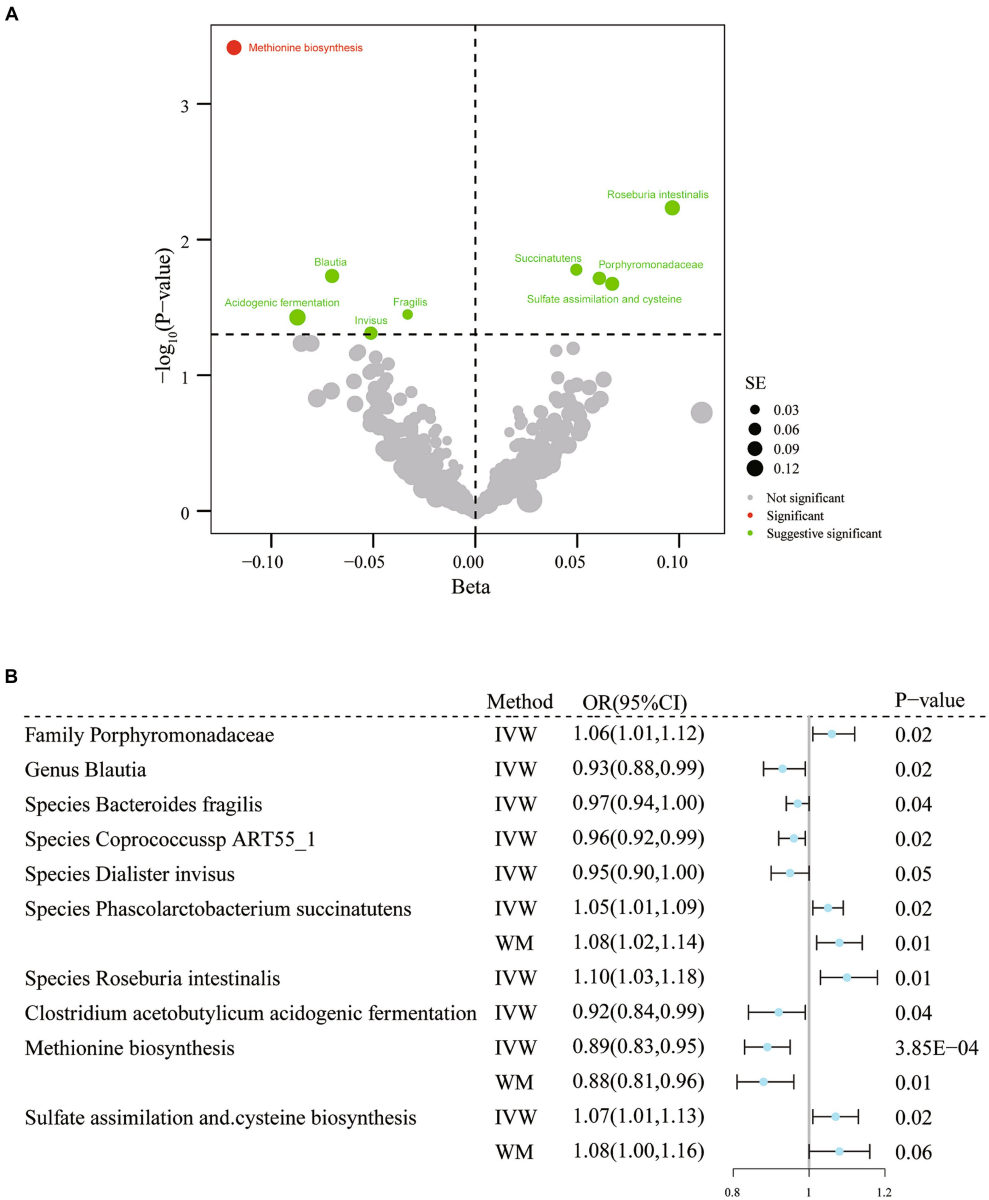
Results of IVW analysis of gut microbiota (Dutch Microbiota Project) and migraine. **(A)** The volcano plot shows the association between 207 gut microbiota and 205 functional pathways with migraine risk. The X-axis represents the β value and the Y-axis shows the logarithmic p-value in base 10. The red dots indicate the *p*-value <0.05 and false discovery rate < 0.05. Green dots indicate *p*-values <0.05 but the false discovery rate > 0.05. **(B)** Forest plot results for association estimation. CI, confidence interval; IVW, inverse-variance weighted; WM, weighted median; OR, odds ratio; MR, mendelian randomization; SE, standard error.

In the preliminary exploration phase, we used the IVW approach to identify eight microbial taxa and three functional pathways that may be associated with migraine (Figure 3; Supplementary Table S5). There was no evidence of weak instrumental variables.

According to the results of IVW analysis, genus *Blautia* (OR = 0.93, 95% CI: 0.88–0.99, *p* = 0.02) and species *Bacteroides fragilis* (OR = 0.97, 95% CI: 0.94–1.00, *p* = 0.04), *coprococcus* sp_ART55_1 (OR = 0.96, 95% CI: 0.92–0.99, *p* = 0.02), and *Dialister invisus* (OR = 0.95, 95% CI: 0.90–1.00, *p* = 0.049) were linked to lower migraine risk. Furthermore, the family *Porphyromonadaceae* (OR = 1.06, 95% CI: 1.01–1.12, *p* = 0.02) and species *Phascolarctobacterium succinatutens* (OR = 1.05, 95% CI: 1.01–1.09 *p* = 0.02) and *Roseburia intestinalis* (OR = 1.10, 95% CI: 1.03–1.18, *p* = 5.84× 10^−3^) were associated with a higher risk of migraine (Figure 3; Supplementary Table S6). Additionally, three related functional pathways include *Clostridium acetobutylicum* acidogenic fermentation (OR = 0.92, 95% CI: 0.84–0.99, *p* = 0.04), methionine biosynthesis (OR = 0.89, 95% CI: 0.83–0.95, *p* = 3.85× 10^−4^), sulfate assimilation, and cysteine biosynthesis (OR = 1.07, 95% CI: 1.01–1.13, *p* = 0.02) were associated with migraine risk (Figure 3; Supplementary Table S6). However, when adjustment for multiplicity was considered, only the association between methionine synthesis (*q* = 0.01) and migraine remained significant; thus, the remaining associations should be considered suggestive (Supplementary Table S6). The association estimates were similar in the sensitivity analyses. No evidence of heterogeneity or horizontal pleiotropy was found (Supplementary Table S6).

### Metabolites and migraine

Based on the pooled data from four human metabolome GWAS, 6 plasma metabolites were found to be associated with migraine (Supplementary Table S7). However, when adjustment for multiplicity was considered, these associations should be considered suggestive (all *q* > 0.05). A search of the gutMGene database revealed that only hydrocinnamate (OR = 0.85, 95% CI: 0.73–1.00, *p* = 0.046) was associated with gut microbial metabolism (Supplementary Table S8). The strength of the included instrumental variables was adequate. Heterogeneity or horizontal pleiotropy was not observed (Supplementary Table S8).

### Reverse MR analysis

When migraine was included as an exposure, the screening threshold was established as 5 × 10^−8^ at the genome-wide level due to the availability of sufficient IVs. The details of the included IVs are provided in Supplementary Table S9. In the reverse MR analysis, there was no significant causal relationship between migraine and the gut microbiota or metabolites (Supplementary Table S10). No evidence of heterogeneity, horizontal pleiotropy, or outliers was found in most of the association analyses (Supplementary Table S10).

## Discussion

The present study employed a two-sample MR design to explore the potential causal relationship between gut microbiota and migraines utilizing the summary statistics for migraine published by the IHGC and for gut microbiota from the MiBioGen or Dutch Microbiota project. According to our MR analysis, *Lachnospiraceae UCG 001* increased the likelihood of migraine, while methionine biosynthesis pathways associated with the microbiota and hydrocinnamate were associated with a reduced migraine risk. Our study also revealed that multiple gut microbiota may be involved in the occurrence and development of migraine. For example, one family (*family XIII*), two genera (*Blautia, Eubacterium* (*nodosa* group)), and three species (*Bacteroides fragilis*, *coprococcus sp_ART55_1*, and *Dialister invisus*) of gut microbiota may have preventive properties against migraine. However, one family (*Porphyromonadaceae*), five genera (*Coprobacter, Hungatella, Marvinbryantia, Parabacteroides, Roseburia*) and two species (*Phascolarctobacterium succinatutens, Roseburia intestinalis*) of gut microbiota may increase the risk of migraine.

Some observational studies have reported a relationship between gut microbiota and migraine (Chen et al., 2019; Georgescu et al., 2019; Geisler et al., 2021; Kopchak et al., 2022; Kopchak and Hrytsenko, 2022; Liu et al., 2022; Miao et al., 2022; Bai et al., 2023; Yong et al., 2023). In the present study, the genus *LachnospiraceaeUCG001*, which had not previously been identified, was discovered to be a risk factor for migraine for the first time. The *Lachnospiraceae* family belongs to the phylum Firmicutes and comprises 58 genera and several unclassified strains, some of which generate butyrate along with various short-chain fatty acids (SCFAs) by hydrolyzing carbohydrates such as starch and sugar (Vacca et al., 2020). Some *Lachnospiraceae* species actively impair glucose metabolism, leading to inflammation (Vacca et al., 2020). The current study showed that *LachnospiraceaeUCG001*, at the genus level, might influence depressive symptoms or major depressive disorder in humans (Jiang et al., 2015; Zheng et al., 2016; Radjabzadeh et al., 2022), and it was also linked to anhedonia in mice at the species level (Yang et al., 2019). Overall, the available evidence has suggested that *LachnospiraceaeUCG001* may affect health, such as in depression and multiple sclerosis. Thus, *LachnospiraceaeUCG001* may adversely affect migraines. Information regarding the physiological role of *LachnospiraceaeUCG001* in neurological disorders is limited and warrants further study.

**Figure 4:**
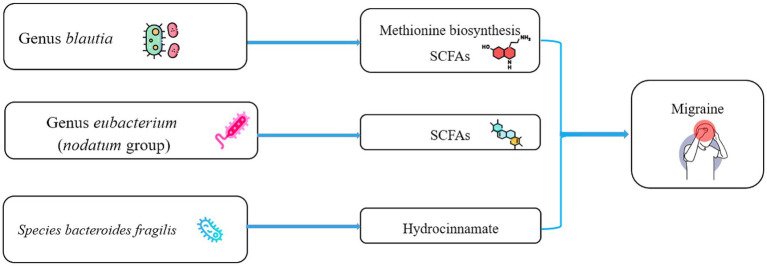
Schematic representation of the potential metabolites of the gut microbiota that influence migraine. SCFAs, short chain fatty acids.

*Blautia* is a genus of anaerobic bacteria with probiotic properties that is dominant in the gut microbiota (Maturana and Cárdenas, 2021). It has the potential to inhibit the colonization of pathogenic bacteria in the intestine and affects the composition of intestinal microbiota (Liu et al., 2021). *Blautia* can use glucose to produce acetic acid, thereby altering the intestinal environment. It may also be related to the metabolism of certain amino acids, such as methionine (Hosomi et al., 2022). Notably, functional pathway analysis of gut microbiota found that methionine synthesis was associated with reduced migraine risk. One of the derived metabolites of *Blautia* is S-adenosylmethionine. S-adenosylmethionine is important in health maintenance and a methyl donor for biological methylation. Recent studies have shown that *Blautia* can help prevent diabetes, obesity, and inflammation and is used to treat depression (Yang et al., 2020; Hosomi et al., 2022). In summary, accumulating evidence reveals that *Blautia* has beneficial effects on human health and can act as a probiotic (Figure 4). *Eubacterium* (*nodatum* group) may play a protective role against migraine, similar to the results of some observational studies (Chen et al., 2019; Mukherjee et al., 2020; Yong et al., 2023). *Eubacterium* can increase the levels of SCFAs, including acetic and butyric acids, and exert anti-inflammatory effects (Figure 4; Mukherjee et al., 2020). The role of *Bacteroides fragilis* in human health is controversial. It has demonstrated both an ability to suppress intestinal inflammation (IL-10) and an increased risk of colorectal cancer (Carrow et al., 2020). However, a search of the gutMGene database suggested that *Bacteroides fragilis* was associated with hydrocinnamate (Jain et al., 2019). Hydrocinnamate has potential antioxidant properties such as scavenging free radicals and activating antioxidant enzymes (Menni et al., 2020). Consistently, our study found a protective effect of genetically predicted higher plasma hydrocinnamate levels against migraine (Figure 4). Therefore, we suggest that *Blautia* and *Eubacterium* (*nodatum* group) at the genus level and *Bacteroides fragilis* at species level may be candidates for migraine treatment in the future. Currently, the literature lacks studies of the effect of *Family XIII*, *coprococcus sp_ART55_1*, and *Dialister invisus* on human health. Thus, it is difficult to judge their potential protective effect. Therefore, there is not enough confidence that these four microbial taxa have an *a priori* protective effect against migraine. One observational study reported a possible negative trend in the correlation between *Prevotellaceae* and migraine duration (Liu et al., 2022). Studies have reported a reduced abundance of *Howardella* at the species at the genus level in individuals with depression (iMSMS Consortium, 2022). However, the causal estimates of the relationship between Prevotellaceae, Eubacterium (rectale group), *Howardella,* and migraine showed inconsistent directions in the IVW and MR-Egger methods (Supplementary Table S3). Therefore, their causal association with migraines needs to be considered with caution in MR analyses.

*Parabacteroides, Hungatella, Coprobacter*, *Marvinbryantia,* and *Roseburia* are potentially positively related to the risk of migraines. In line with the present findings, the abundance of *Parabacteroides* was higher in patients with migraines than controls, and there was a possible positive correlation between *Parabacteroides* and Pittsburgh Sleep Quality Index score (Bai et al., 2023; Yong et al., 2023). *Parabacteroides* belongs to the phylum *Bacteroidetes*, a group of Gram-negative anaerobic bacteria that regularly colonize the human digestive system. Lipopolysaccharides and the metabolic end-product succinate are two ways that *Parabacteroidetes* cause inflammation (Ezeji et al., 2021). Observational studies have found a greater abundance of *Hungatella* in patients with chronic migraine compared to people without migraine (Yong et al., 2023). Studies have reported that the abundance of Hungatella was higher in patients with multiple sclerosis compared to controls and was positively associated with depressive symptoms (iMSMS Consortium, 2022; Radjabzadeh et al., 2022). *Hungatella* is a member of the family *Clostridiaceae* and the phylum Firmicutes. *Hungatella* produces the precursor molecule trimethylamine-N-oxide, which has been linked to neurological disorders like depression (Janeiro et al., 2018). Some studies have reported that the abundance of *Coprobacter* was higher in parents with neurosyphilis, general paresis, or sarcopenia, as well as kids suffering from autism spectrum disorder, compared with controls (Liu et al., 2019; Wang Y. et al., 2022; Wang G. et al., 2022). *Coprobacter* has a potential pathogenic role that may be related to the elevation of trimethylamine-*N*-oxide; further investigation should be done to better comprehend its function in the pathogenesis of migraine (Shirouchi et al., 2022).

In the reported observational studies, *Marvinbryantia* was positively correlated with vitamin D3, and *Marvinbryantia* abundance was inversely correlated with the likelihood of amyloid positivity (Zuo et al., 2019; Verhaar et al., 2021). This implies that *Marvinbryantia* is a beneficial genus. *Marvinbryantia*, which belongs to the phylum Firmicutes, is a cellulose-degrading bacterial genus. On the one hand, *Marvinbryantia* can produce acetate in the intestine, thereby promoting butyrate synthesis. In contrast, Gordon et al. found that *Marvinbryantia* can improve succinate production *in vivo* (Rey et al., 2010). By regulating IL-1β expression and reactive oxygen species generation, succinate has an effect on key inflammatory pathways in immune and non-immune cells (Lampropoulou et al., 2016). *Roseburia* is in the phylum Firmicutes and family Lachnospiraceae. Five well-characterized species of the *Roseburia* genus, including the *Roseburia intestinalis*, *Roseburia hominis*, *Roseburia inulinivorans*, *Roseburia faecis*, and *Roseburia cecicola*, all produce SCFAs (Nie et al., 2021). In addition, *Roseburia* can convey messages to colonic glia to trigger IL-22 release and influence 5-hydroxytryptamine levels and glial fibrillary acidic protein expression in colonic tissue (Peh et al., 2022). Therefore, *Roseburia* exert potential beneficial effects on the pathogenesis of neurological diseases like stroke, depression, and Parkinson’s disease (Nie et al., 2021; Peh et al., 2022). However, the present study revealed that *Roseburia* may increase migraine risk. One study found that *Roseburia* was less abundant at the genus level in people with migraine compared to those without (Yong et al., 2023). However, a mouse model of migraine did not reveal any significant differences (Nan et al., 2023). In this context, the relationship between *Roseburia* and migraines should be viewed cautiously and warrants further exploration.

As discussed previously, *Eubacterium* can increase levels of SCFAs and exert anti-inflammatory effects. The end-products of the metabolism of the human intestinal flora are SCFAs, which mainly contain acetic acid, propionic acid, and butyric acid. The SCFA-producing bacterial groups identified in this study as being associated with migraine were *Blautia* and *Eubacterium* (*nodatum* groups). Studies have reported that SCFAs can decrease microglial activation and pro-inflammatory cytokine secretion, such as mitogen-activated protein kinases, nuclear factor-κB, IL-1β, and TNF-α (Dalile et al., 2019; Silva et al., 2020). SCFAs affect inflammatory signaling pathways mainly by activating transmembrane G protein-coupled receptors, such as olfactory receptor 51E2, GPR109A, GPR41, and GPR42 (Dalile et al., 2019). For example, propionate and butyrate reduced the onset of pain in a nitroglycerine-induced mouse model of migraine and reduced the release of IL-1β and TNF-α in the ileum (Lanza et al., 2021). SCFAs regulate neurotransmitter and neurotrophic factor levels. For example, the neurotransmitters glutamate, glutamine, and γ-aminobutyric acid are altered by acetate in hypothalamus (Frost et al., 2014). Propionate and butyrate affect intracellular potassium levels and thus participate in the operation of cellular signaling systems (Oleskin and Shenderov, 2016). SCFA can also regulate tryptophan and tyrosine metabolism and affect serotonin and dopamine levels (Clarke et al., 2014). In a rat model of migraine known as the inflammatory soup model, it was found that bacteria associated with the production of SCFAs and 5-hydroxytryptamine were reduced, resulting in decreased levels of 5-hydroxytryptamine and tryptophan hydroxylase (Nan et al., 2023). Additionally, SCFAs, especially propionate and butyrate, have been found to inhibit the activity of histone deacetylases, thereby regulating the acetylation of histone lysine residues in nucleosomes and protecting nerve cells.

The findings of two randomized controlled trials suggested that supplementation with probiotics, specifically *bifidobacterium*, *lactobacillus*, and *lactococcus* strains, could potentially decrease the frequency of migraine. However, another randomized controlled trial did not support this claim (De Roos et al., 2017). Further, three studies did not find that probiotic supplementation could significantly improve intestinal permeability or reduce the secretion of inflammatory factors (Martami et al., 2019; Rinninella et al., 2019; Ghavami et al., 2021). However, the present study did not identify a potential causal relationship between *Bifidobacterium*, *Lactobacillus,* or *Lactococcus* and migraine.

This study has multiple strengths compared to previous studies. First, we complemented the gap in the species-level relationship between gut microbiota and migraine. Second, we identified three gut microbial taxa that serve as beneficial bacteria and show promise as candidate therapeutic targets for migraine treatment. Third, our findings revealed that a functional pathway for methionine synthesis associated with the gut microbiota is associated with a reduced risk of migraine. Fourth, we identified a plasma metabolite (hydrocinnamate) that may act as a hub between gut microbiota and migraine.

Nevertheless, this study had some limitations. First, to include more genetic variation in IVs, the significance level for gut microbiota selection of IVs was set at 1 × 10^−5^, rather than using the traditional GWAS significance threshold (*p* < 5 × 10^−8^). However, F-statistics >10 can rule out potential weak instrument bias, and FDR correction was used to limit the possibility of false positives. Second, some of the microbiota found to be associated with migraine were not successfully replicated in a different dataset. However, we provide possible directions for exploration that warrant further discussion regarding their true relationship with migraine. Third, the low statistical power of some association analyses should not be ignored. Fourth, since the sample size of the gut microbiota was relatively small, weak instrumental bias may have affected the reverse MR analysis; therefore, reverse causality could not be completely ruled out. Fifth, because the majority of the GWAS participants used as our gut microbiota data were of European heritage, our findings may not be generalizable to all populations. Further studies on the causal relationship between the gut microbiota and migraine in different global regions should be considered to obtain better generality. Sixth, given that the analysis used summary statistics, subgroup analyses according to migraine subtype and sex were not performed. Seventh, direct mechanistic studies supporting our findings are still lacking, and more studies are needed to gain more direct proof of any proposed connection.

## Conclusion

This bidirectional two-sample MR study concluded that there is a causal relationship between *Lachnospiraceae UCG 001* and migraine. However, further studies are required to clarify the underlying pathogenic mechanisms. Additionally, genus *Blautia*, genus *Eubacterium* (*nodatum* group), species *Bacteroides fragilis*, the methionine biosynthesis pathway related to gut bacteria, and hydrocinnamate were found to be protective factors against migraine. These findings provide potential targets for migraine intervention. Migraine and gut microbiota do not appear to be causally related; however, inverse MR analysis was unable to entirely rule out the effects of migraine on gut microbiota.

## Data availability statement

The original contributions presented in the study are included in the article/Supplementary material, further inquiries can be directed to the corresponding author.

## Author contributions

KQ: Conceptualization, Formal analysis, Methodology, Software, Writing – original draft. M-XL: Conceptualization, Methodology, Writing – original draft. LG: Formal analysis, Software, Writing – original draft. Z-TC: Visualization, Writing – original draft. J-JL: Investigation, Writing – original draft. RY: Investigation, Writing – original draft. MD: Data curation, Project administration, Supervision, Writing – review & editing.

## Footnotes

^1^ www.phenoscanner.medschl.cam.ac.uk

^2^ http://cnsgenomics.com/shiny/mRnd
